# Supplementary material for: Degradation of Human Serum Albumin by Infrared Free Electron Laser Enhanced by Inclusion of a Salen-Type Schiff Base Zn (II) Complex
Source: Int J Mol Sci. 2020 Jan 29;21(3):874. doi: 10.3390/ijms21030874 (PMC7037271; doi:10.3390/ijms21030874)
Supplement: Supplementary file 1 [file ijms-21-00874-s001.pdf]

SUPPLEMENTARY MATERIAL

Table S1. Comparison of the calculated harmonic wavenumbers (ν^{cal}, cm^{-1}), absolute (A) and relative infrared intensities (A, %), reduce masses (μ), force constants (f, mDyne/Å), scaled wavenumbers (ν^{scal}, cm^{-1}), experimental wavenumbers by IR (ν^{exp}), and characterization obtained in the ZnL molecule at the B3LYP/6-31G(d,p) level.

Theoretical						Experimental	
ν^{cal}	A	A^{a}%	μ	f	$\nu^{scal,\,b}$	ν^{exp}	Characterization
433.9	31	2	4.3	0.47	429	426 vw	60% γ(C-N)
462.2	28	2	5.7	0.72	457		70%, δ(ZnO_2N_2 group)
462.6	1	0	4.8	0.60	457	458 w	40%, δ(ZnO_2N_2 group)
476.4	11	1	4.2	0.56	471	472 w	43%, δ(ZnO_2N_2 group) +30%, 16b γ(CCC) in benzene ring
484.7	3	0	3.6	0.50	479	495 vw	54%, 16b γ(CCC) in benzene ring
552.2	1	0	3.4	0.61	545	536 w	63%, 16a γ(CCC) in benzene ring
554.3	3	0	3.4	0.61	547		63%, 16a γ(CCC) in benzene ring
556.0	6	0	4.8	0.87	549	553 w	60% γ(O-Zn-O) + 20%, δ(CCC) in benzene ring
559.4	6	0	4.9	0.90	552	564 w	60% γ(O-Zn-O) + 20%, δ(CCC) in benzene ring
598.1	2	0	5.6	1.19	590	594 m	70% δ_s(O-Zn-O) + 20%, 6a δ(CCC) in benzene ring
610.2	67	5	8.3	1.82	602	588 m	73% δ_{as}(O-Zn-O) + 20%, 6a δ(CCC) in benzene ring
624.7	2	0	5.7	1.30	616	618 m	43% δ(O-Zn-O) + 30%, 6a δ(CCC) in benzene ring
652.2	9	1	5.6	1.41	643	646, 640 w	60%, 6a δ(CCC) in benzene ring
676.8	3	0	4.4	1.19	667	671 m	40%, δ(C-C-N) + 30%, 6a δ(CCC) in benzene ring
751.0	2	0	2.9	0.97	740		83%, 6b γ(CCC) in benzene ring
752.2	0	0	2.9	0.98	741	739 m	83%, 6b γ(CCC) in benzene ring
766.6	66	5	1.4	0.48	756	749 s	95%, 11 γ(C-H) in benzene ring
766.7	23	2	1.4	0.48	756		95%, 11 γ(C-H) in benzene ring
773.3	2	0	1.3	0.48	762	761 m	65% γ(C-H) in $C36H_2$ + 30% γ(C-H) in $C30H_2$, $C33H_2$
795.8	0	0	4.6	1.71	784		35% ν(O-Zn-O) + 25% δ(CCO)+ 20% δ(CCC) in benzene ring
804.6	17	1	4.4	1.67	793	793 m	35% ν(O-Zn-O) + 25% δ(CCO)+ 20% δ(CCC) in benzene ring
852.8	0	0	3.5	1.50	840	844 w	60% δ(C-N) + 25% δ(O-Zn-O)
870.9	10	1	1.6	0.71	858	850 m	90%, 17b γ(C-H) in benzene ring
871.4	4	0	1.6	0.72	858	857 w	90%, 17b γ(C-H) in benzene ring
914.5	60	5	5.6	2.77	901	905 m	60%, 12 δ(CCC) + 30%, δ(C-O)
920.3	3	0	4.9	2.45	906		60%, 12 δ(CCC) + 30%, δ(C-O)
942.3	1	0	1.3	0.68	928	937 m	85%, 17a γ(C-H) in benzene ring
942.7	0	0	1.3	0.68	928		85%, 17a γ(C-H) in benzene ring
968.5	9	1	2.0	1.08	954	942 vw	95% γ(C-H) in CH_2
991.4	0	0	1.3	0.74	976	969 vvw	85%, 5 γ(C-H) in benzene ring
991.4	0	0	1.3	0.74	976		85%, 5 γ(C-H) in benzene ring
1002.2	7	1	1.6	0.97	987	982 w	70%, γ(C-H) in C9H, C15H
1010.6	10	1	1.5	0.92	995	995 vw	70%, γ(C-H) in C9H, C15H
1020.1	5	0	2.3	1.42	1004	1012 w	40%, ν(C30-N, C33-N) +30% ν(C=N) +δ(C-H) in benzene ring
1055.4	10	1	2.2	1.48	1039	1036 m	85%, 18b δ(C-H) in benzene ring
1056.3	1	0	2.2	1.44	1040	1040 w	85%, 18b δ(C-H) in benzene ring
1098.5	5	0	3.9	2.80	1081	1071 w	60%, ν(C30-N, C33-N) + 25% δ(C-H) in CH_2
1113.0	3	0	2.2	1.57	1095	1118 w	60%, ν(C-C30-C)
1123.4	36	3	3.3	2.42	1106	1131 vw	60%, ν(C30-N, C33-N) + 25% δ(C-H) in CH_2
1157.6	32	2	1.6	1.29	1139	1137 m	85%, 15 δ(C-H) in benzene ring
1161.4	5	0	1.7	1.33	1143		85%, 15 δ(C-H) in benzene ring
1181.7	16	1	1.1	0.92	1163		90%, 9a δ(C-H) in benzene ring
1183.0	44	3	1.1	0.93	1164	1157 m	90%, 9a δ(C-H) in benzene ring
1229.6	49	4	2.3	2.01	1210	1202 s	80%, 9b δ(C-C, C-H) in benzene ring

1230.6	20	2	2.4	2.17	1211	1212 m	80%, 9b δ(C-C, C-H) in benzene ring
1253.6	25	2	1.3	1.16	1233	1231 vw	65% δ(C-H) in C30H_2, C33H_2 + δ(C-H) in benzene ring
1255.0	27	2	1.2	1.14	1235	1244 vw	65% δ(C-H) in C30H_2, C33H_2 + δ(C-H) in benzene ring
1275.7	7	1	1.6	1.55	1255	1260 w	85% δ(C-H) in CH_2
1278.6	14	1	1.6	1.54	1258	1271 w	70%, 3 δ(C-H) in benzene ring
1300.0	4	0	1.1	1.12	1279	1277 vw	53% δ_s(C-H) in C30H_2, C33H_2 + 45% δ_s(C-H) in C36H_2
1364.4	22	2	2.0	2.23	1342	1310 s	60% δ_s(C-H) in C30H_2, C33H_2 + 15% δ_s(C-H) in C36H_2
1369.5	92	7	1.7	1.83	1347	1324 w	50% δ_s(C-H) in C30H_2, C33H_2 + 35% δ_s(C-H) in C36H_2
1371.6	0	0	4.0	4.44	1349	1346 sh	50%, ν(C-O) + 40%,19b ν(C=C) in benzene ring
1380.3	58	4	1.5	1.66	1358	1340 m	65% δ_s(C-H) in C36H_2 + 30% δ_s(C-H) in C30H_2, C33H_2
1400.2	35	3	1.3	1.47	1377	1351 sh	70% δ_s(C-H) in C30H_2, C33H_2 + 25% δ_s(C-H) in C36H_2
1400.6	72	6	3.2	3.71	1378	1365 w	80%, 14 ν(C=C) +δ(C-H) in benzene ring
1404.6	1	0	2.9	3.32	1382		80%, 14 ν(C=C) +δ(C-H) in benzene ring
1436.5	109	8	1.6	1.89	1413	1400 m	70%, δ(C-H) in C9H, C15H
1438.8	1	0	1.6	1.94	1415	1423 m	70%, δ(C-H) in C9H, C15H
1489.2	120	9	2.9	3.84	1439	1449 s	50%,19b ν(C=C)+δ(C-H) in benzene ring +30% δ(C-H) in C30H_2,C33H_2
1494.6	44	3	1.2	1.56	1444		60%, δ_{as}(C-H) in C36H_2 + 20% δ_{as}(C-H) in C30H_2, C33H_2
1505.8	77	6	2.2	2.99	1455		60%, δ_{as}(C-H) in C36H_2
1505.9	63	5	2.1	2.77	1455		70%, 19a ν(C=C)+ δ(C-H) in benzene ring + δ(C-H) in C36H_2
1507.2	64	5	2.2	2.88	1456		70%, 19a ν(C=C)+ δ(C-H) in benzene ring + δ(C-H) in C36H_2
1512.5	119	9	1.3	1.80	1461	1475 vs	85%, δ_{as}(C-H) in CH_2
1517.1	38	3	1.4	1.93	1465		85%, δ_{as}(C-H) in CH_2
1576.2	166	13	4.9	7.21	1521	1548 vs	80%, 8b ν(C=C) in benzene ring
1577.4	125	10	5.1	7.43	1522		80%, 8b ν(C=C) in benzene ring
1666.4	102	8	5.7	9.28	1606	1575 vs	70%, 8a ν(C=C) in benzene ring
1668.6	10	1	5.7	9.42	1608	1605 m	70%, 8a ν(C=C) in benzene ring
1683.8	1308	100	6.5	10.81	1622	1628 vs	78%, ν(C9=N, C15=N)
1701.2	190	15	6.8	11.62	1639	1647 vs	80%, ν(C9=N, C15=N)
						1738 m	ν(CO)
						2364,2356vs	ν(C-H ··O) intermolecular
3017.1	32	2	1.1	5.71	2880	2862 vw	100%, ν(C-H)
3017.5	55	4	1.1	5.71	2880	2875 vw	100%, ν(C-H)
3040.9	46	4	1.1	5.92	2902		100%, ν(C-H)
3041.5	35	3	1.1	5.92	2903	2910 w	100%, ν(C-H)
3048.1	75	6	1.1	5.81	2909	2918 w	100%, ν(C-H)
3069.8	6	0	1.1	6.11	2929	2931 sh	100%, ν(C-H)
3079.3	12	1	1.1	6.15	2938	2940 s	90%, ν_{as}(C-H) in C30H_2, C33H_2
3099.4	56	4	1.1	6.25	2957	2970 m	70%, ν_{as}(C-H) in C36H_2 + 30%,ν_{as}(C-H) in C30H_2, C33H_2
3162.3	15	1	1.1	6.41	3016		92%, ν(C-H) in H10, H22 of benzene ring
3162.4	11	1	1.1	6.41	3017		92%, ν(C-H) in H10, H22 of benzene ring
3178.2	13	1	1.1	6.48	3031		100%, ν(C-H)
3178.2	17	1	1.1	6.48	3031	3037 m	95%, 20b ν(C-H) in benzene ring
3207.3	37	3	1.1	6.62	3059	3051 m	95%, 20b ν(C-H) in benzene ring
3207.4	5	0	1.1	6.62	3059		100%, ν(C-H)
3213.0	22	2	1.1	6.67	3064	3099 vw	100%, ν(C-H)
3213.1	11	1	1.1	6.67	3064		100%, ν(C-H)

[a]Normalized to the highest value. [b]With the scaling equation: $\nu^{scal} = 3.3+0.9813\ \nu^{cal.}$ (for the 400-1450 cm^{-1} range) and $\nu^{scal} = 34.7+0.9429\ \nu^{cal.}$ (for the 1450- 3300 cm^{-1} range).
